# Natural compound targeting *BDNF* V66M variant: insights from in silico docking and molecular analysis

**DOI:** 10.1186/s13568-023-01640-w

**Published:** 2023-11-28

**Authors:** Azra Sakhawat, Muhammad Umer Khan, Raima Rehman, Samiullah Khan, Muhammad Adnan Shan, Alia Batool, Muhammad Arshad Javed, Qurban Ali

**Affiliations:** 1 Institute of Molecular Biology and Biotechnology, The University of Lahore, Lahore, Pakistan; 2 Centre for Applied Molecular Biology, University of the Punjab, Lahore, Pakistan; 3 Department of Plant Breeding and Genetics, Faculty of Agricultural Sciences, University of the Punjab, Lahore, Pakistan

**Keywords:** Brain derived neurotropic factor, Therapy, Valine, Methionine

## Abstract

*Brain-Derived Neurotrophic Factor *(*BDNF*) is a neurotrophin gene family gene that encodes proteins vital for the growth, maintenance, and survival of neurons in the nervous system. The study aimed to screen natural compounds against *BDNF* variant (V66M), which affects memory, cognition, and mood regulation. *BDNF* variant (V66M) as a target structure was selected, and Vitamin D, Curcumin, Vitamin C, and Quercetin as ligands structures were taken from PubChem database. Multiple tools like AUTODOCK VINA, BIOVIA discovery studio, PyMOL, CB-dock, IMOD server, Swiss ADEMT, and Swiss predict ligands target were used to analyze binding energy, interaction, stability, toxicity, and visualize *BDNF*-ligand complexes. Compounds Vitamin D3, Curcumin, Vitamin C, and Quercetin with binding energies values of − 5.5, − 6.1, − 4.5, and − 6.7 kj/mol, respectively, were selected. The ligands bind to the active sites of the *BDNF* variant (V66M) via hydrophobic bonds, hydrogen bonds, and electrostatic interactions. Furthermore, ADMET analysis of the ligands revealed they exhibited sound pharmacokinetic and toxicity profiles. In addition, an MD simulation study showed that the most active ligand bound favorably and dynamically to the target protein, and protein–ligand complex stability was determined. The finding of this research could provide an excellent platform for discovering and rationalizing novel drugs against stress related to *BDNF* (V66M). Docking, preclinical drug testing and MD simulation results suggest Quercetin as a more potent *BDNF* variant (V66M) inhibitor and forming a more structurally stable complex.

## Introduction

*Brain-Derived Neurotrophic Factor (BDNF)* is a neurotrophin gene family gene that encodes proteins vital for the growth, maintenance, and survival of neurons in the nervous system. It produces the BDNF protein, which regulates neuron survival, development, mood modulation, synapse formation, neurotransmitter balance, and neuroprotection. Therefore, it is crucial to studying neurobiology and neuroscience due to their broad impact on the nervous system's growth and operation (Ibrahim et al. 2022).

The altered or defective BDNF protein that results from *BDNF* gene mutations affects the nervous system's growth and operation (Azoulay et al. 2019). Alzheimer's disease, depression, schizophrenia, and other cognitive diseases are only a few of the neurological and psychiatric conditions for which *BDNF* gene alterations have been researched (Gao et al. 2022; Lima Giacobbo et al. 2019). The economic impact of mental illness in 2010 was estimated to be a loss of 2.5 trillion dollars (USD) in global economic output, with an anticipated loss of 16.3 trillion dollars (USD) from 2011 to 2030. It is also one of the leading causes of years lived with impairments across all disease groups. Mental illness is a significant public health concern affecting millions worldwide (Alvi et al. 2020; Organization 2021).

*BDNF* has been found in several brain regions, including the hippocampus, hypothalamus, amygdala, and neocortex, which are crucial for emotional regulation. *BDNF* supports various signaling pathways, such as cholinergic, dopaminergic, and serotonergic. A correlation has been established between the development of major depressive disorder (MDD) and the *BDNF* genotype. Furthermore, the Val66Met polymorphism, which is present in the human chromosome 11p13, has been linked to a hereditary susceptibility to anxiety and depression in animals. The substitution of valine with methionine at position 196 in exon 2 due to a non-synonymous G to A single-nucleotide polymorphism (Val66Met) has garnered the most attention among multiple known significant gene polymorphisms (Khan et al. 2019; Wang et al. 2023).

The Neurotrophic Receptor Tyrosine Kinase 2 (NTRK2) is the receptor for Brain-derived Neurotropic Factor (*BDNF),* a protein that plays a crucial role in the molecular pathways of brain plasticity. When BDNF binds to NTRK2, it activates the MAPK pathway, which controls synaptic plasticity and repair. *BDNF* is, therefore, essential for various cognitive functions, such as memory formation, long-term potentiation, and the growth and differentiation of the central nervous system (Purves et al. 2020).

The *BDNF* gene has a common single-nucleotide polymorphism (SNP) called rs6265, which replaces Valine (Val) with Methionine (Met) at amino acid position 66. This change leads to a reduction in the amount of BDNF protein produced. In healthy individuals, this substitution affects motor learning, cognition, and memory, and it can also impact the severity of cognitive impairment, motor ability, and recovery from neurological damage (Shen et al. 2018; Volf et al. 2023). This SNP, commonly known as the Val66Met polymorphism, has been thoroughly investigated for its potential relationship with cognitive function and brain illnesses. The Val66Met polymorphism has been associated with *BDNF* secretion and function variations, which may affect memory, mood regulation, and brain plasticity (Brown et al. 2020; Tian et al. 2021). However, the precise effects of this polymorphism are complex and subject to various genetic and environmental influences. The impact of the Val66Met polymorphism and other *BDNF* variants on brain function and associated illnesses is still being researched (Devlin et al. 2021). In the endoplasmic reticulum, pre-pro-*BDNF*, a precursor protein made during BDNF production, is converted into pro-B*DNF* (da Fonseca et al. 2021; De Oliveira et al. 2019). Pro-BDNF may undergo cleavage into mature *BDNF* (m-*BDNF*) within vesicles in the trans-Golgi network. All cell types release dimeric pro-*BDNF* and dimeric m-*BDNF* through constitutive release or activity-dependent exocytosis in excitable cells that secrete granules. The released homodimers bind to their related receptors, tropomyosin kinase (Trk) and p75 neurotrophin receptor, triggering several intracellular signaling cascades linked to proliferation, maturation, and maintenance of neuronal function (Conroy and Coulson 2022; Kowiański et al. 2018).

A multitude of disease-causing mutations can profoundly impact protein structure and function. Proteins are crucial for several bodily functions, and mutations can change stability, acltivity, or interactions with other molecules. These modifications might disrupt enzymatic processes, structural processes, or signaling pathways, which can ultimately result in the development of disease (Goldenzweig and Fleishman 2018; Rodrigues et al. 2018). In contrast to X-ray crystallography, cryo-EM does not require protein crystallization. Instead, it entails using an electron microscope to image frozen samples. Cryo-EM has grown in popularity recently because it can handle complex targets and has fewer sample needs (Chojnowski et al. 2022; García-Nafría and Tate 2021). Researchers can now predict protein structures using computer modeling, owing to improved computational techniques and increasing computing power. To infer protein structures from sequence data, methods like homology modeling and ab initio structure prediction have been applied (Prior et al. 2020; Torrisi et al. 2020). Although these techniques are not as precise as the experimental ones, they can still offer important insights when gathering experimental data is impossible or difficult. The involvement of BDNF in brain development, plasticity, and survival has made the Val66Met polymorphism particularly interesting in several neurological and psychiatric diseases. The following psychological conditions and cognitive functions have been researched concerning this polymorphism **(**Haider et al. 2023**)**.

The potential linkages between neurodegenerative conditions such as Alzheimer's disease and the Val66Met polymorphism in *BDNF* are crucial for the survival and maintenance of neurons. The polymorphism may influence the development or susceptibility of certain illnesses, including an increased incidence of mood disorders, especially depression and bipolar disorder. In regions of the brain associated with emotion processing, *BDNF* plays a role in mood regulation and is vital for neuronal plasticity. Although research has examined the connection between schizophrenia, a complex psychiatric condition, and the Val66Met polymorphism, it is limited, and more studies are needed to understand the impact of the polymorphism on synaptic plasticity and cognitive processes in schizophrenia (Baig et al. 2021; Di Carlo et al. 2019). *BDNF* plays a key role in these processes. According to a previous study, individuals with the Met variant might exhibit memory and cognitive flexibility variations. It is crucial to emphasize that a complex association exists between the Val66Met polymorphism and these brain illnesses. While several studies have found links between the Val66Met polymorphism and specific disorders, the findings have been inconsistent and occasionally contradictory across populations (Grant et al. 2018; Medaglia et al. 2018; Saif et al. 2023).

Treatments for controlling those issues might be made available if a *BDNF* gene mutation is linked to a neurological or psychiatric illness such as schizophrenia or depression (Kular et al. 2018; Niazi et al. 2023). These therapies could consist of drugs, psychotherapy, or other interventions for the patient (Petrosino et al. 2021; Sánchez-Lanzas and Castaño 2021).

Therefore, exploring BDNF and its genetic variations as potential therapeutic targets for neurodegeneration, mood disorders, and cognitive impairment. Additionally, investigating the impact of phytochemicals found in many plant-based foods on *BDNF* expression and function could provide neuroprotective benefits. Phytochemicals such as vitamin D3, curcumin, quercetin, and vitamin C are present in fruits and vegetables and may boost *BDNF* expression, counteracting the reduced secretion caused by the Met allele.

Psychological disorders are increasing in Pakistan, and *BDNF* variant (V66M) has been associated with it globally. Therefore, it is essential to determine the functional role of *BDNF* variant (V66M) in psychological disorders in Pakistan. So, this in silico study aimed to screen natural phytochemical compounds against a *BDNF* variant (V66M).

## Methodology

### Dataset of target peptide

Sequences and information about *BDNF* and its naturally occurring variant V66M were gathered from the UniProt (UniProt ID: P23560), OMIM (OMIN ID: 113505), and PubMed databases. The highest resolution *BDNF* crystallographic structure from the Protein Data Bank (PDB) database (PDB ID: 1BND**)** was chosen as the modeling reference.

### Dataset of natural compound

We have selected four compounds for docking from Pubchem (Štekláč et al. 2021), including Quercetin Compound CID: (5280343), Curcumin Compound CID: (969516), Vitamin D3 Compound CID: (5280795) and Vitamin C Compound CID: (54670067).

### Ligand preparation

The compounds' three-dimensional (3D) structures of the compounds were created using PubChem and exported to PyMOL software user interface. The improved ligands were saved in Protein Data Bank (PDB) file format and then exported into the AutoDock Vina interface, where they underwent additional preparation and were saved in the PDBQT file format.

### Protein preparation

BDNF and its naturally occurring variant, V66M, were obtained from UniProt and PubMed databases. The functional and stability prediction of the 3D crystal structure of the BDNF variant (V66M) was analyzed using the following algorithms: The linked ligands, water molecules, and other heteroatoms were eliminated once exported into the Discover Studio software interface. The prepared protein was then exported into the AutoDock Vina interface, where the missing atoms were examined and mended, Kollman's charges were inserted, and polar hydrogen was dissolved. It was then saved in PDBQT file format.

### Molecular docking analysis

Molecular docking involves examining the interactions between two molecules to determine the best way for them to bind to one another and form a stable complex. AUTODOCK VINA (Krause et al. 2023) was used for docking and predicting the docking score, structural interaction, and stability between the Peptide *BDNF* (Val66Met) and four different compounds. The peptide ligand docking model interaction was used to calculate the energy scores and breakdown of the free energy. The PDB for the top-ranked docked model complex was then visualized using the PyMOL and Discovery studio tools, and the most precise structure was chosen using the docking score or binding energy.

### Identification of binding cavity detection of *BDNF* variant V66M

CB-dock online server (Sh 2022) was used to identify the binding cavity of the human *BDNF* variant V66M. Using the CB-Dock protein–ligand docking method, binding cavity sites are automatically recognized, their central location and dimension have been determined, and the docking box measurement has been altered to allow input ligands.

### Pre-clinical testing of drug analyses/toxicity analyses

The top-ranked compounds underwent toxicity evaluations using the Swiss ADMET server (Adane et al. 2021). ADMET server was used to investigate selected compounds' absorption, distribution, metabolism, excretion, and toxicity.

### Molecular dynamic simulation

The iMODS software was utilized to conduct deformability and Eigenvalue analyses on the MDS complexes. These analyses aimed to identify any residues that might still be unstable or distorted following the coarse-grained MDS (Rehman et al. 2022). The bioactivities of protein structures are influenced by their dynamics. However, it is frequently difficult to investigate protein flexibility through wet laboratory studies, necessitating in-silico methods. Using coarse-grained simulation models in conjunction with the reconstruction of predicted structures to all-atom representation is an efficient computational strategy for exploring protein flexibility in a biological system. The CABSflex 2.0 server was used in this investigation to do molecular dynamics (MD) simulations of the complex of the various ligands, including Vitamin D3, Curcumin, Vitamin C, and Querectin with *BDNF* variation (V66M). Using the "browse" option, the PDB file for the complex was uploaded to the server. CABS-flex uses a set of simulation settings and distance restrictions. These parameters were chosen to offer the highest probability of convergence between CABS-flex simulations and the consensus interpretation of globular protein fluctuations in an aqueous solution. The root-mean-square fluctuation (RMSF) of the protein structure was used to represent the outcomes of the MD simulations. RMSF is the root-mean-square-deviation time average calculated using Eq. (1) as follows:

1 $${\text{RMSF}} = \surd \left( { < xi - < xi } \right)$$

where *xi* is the coordinate of the particle *i,* and <x*i*> is the ensemble average position of *i.*

## Results

**Fig. 1:**
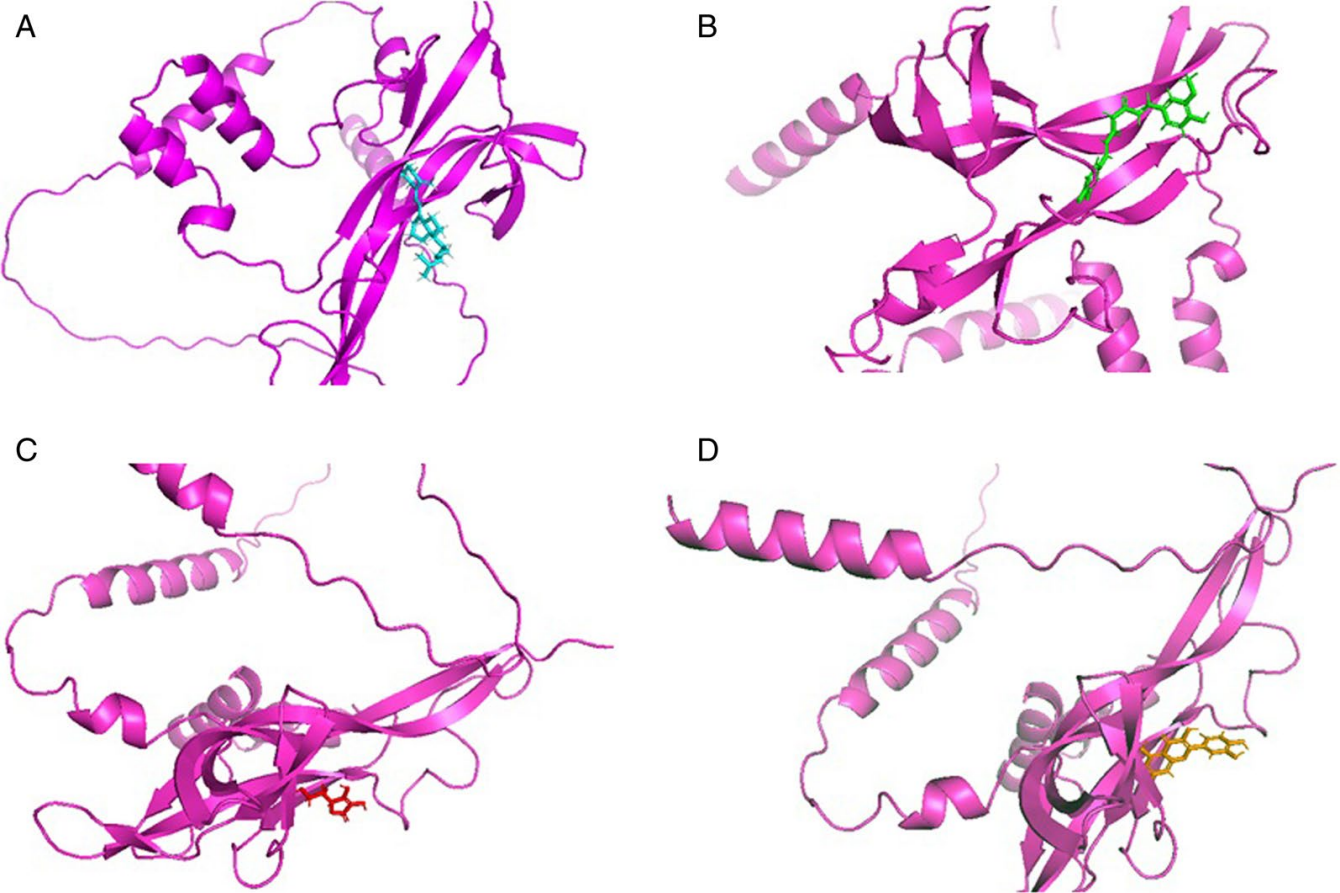
In silico modele structure cartoon representation of 3D *BDNF* variant V66M with different natural compounds complex. **A** Cartoon represents 3D Crystal structure of *BDNF* (V66M) light pink color with ligand Vitamin D3 in cyan color. **B** Curcumin in green color. **C** Vitamin C in red color. **D** Quercetin in brown color

This study aimed to digitally screen the human *BDNF* variation V66M and conduct in *silico* analysis to create, find, and assess powerful natural compounds used as inhibitors. Molecular docking investigations of the selected libraries revealed discrepancies in the observed binding energies. The most effective pose with the lowest binding energy was identified for each molecule. According to the data gathered, the top ranked of the four compounds, one from each selected Pubchem, efficiently bonded to the human *BDNF* variant V66M (Fig. 1).

### Molecular docking of multiple ligands with *BDNF* variant (V66M)

**Fig. 2:**
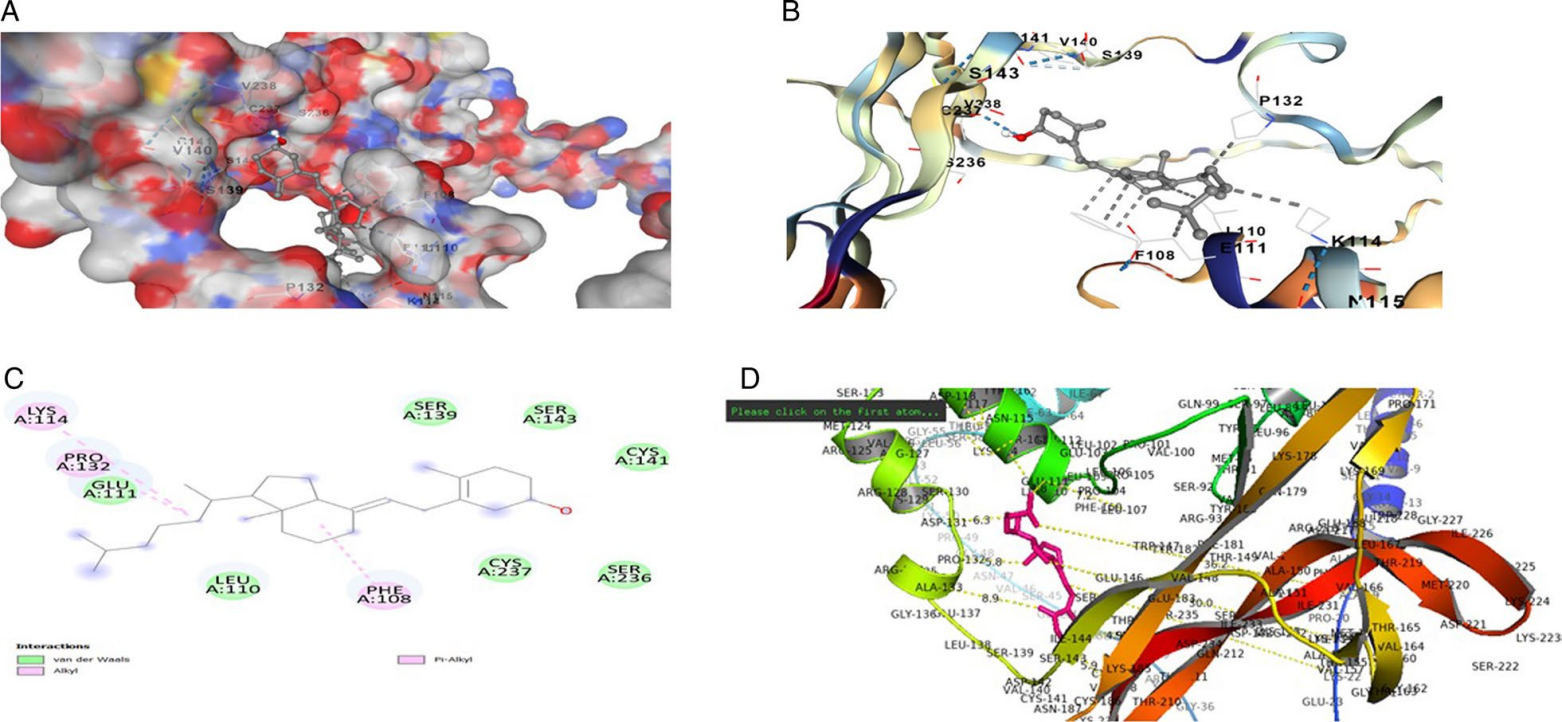
In silico modeled structure cartoon representation of complex *BDNF* variant V66M/Vitamin D3. **A** Spherical cartoon structure represent the binding pocket cavity interacting of residue between *BDNF* residue with Vitamin D3. **B** Shown interacting residue of *BDNF* muted Val-140, cysteine-37, proline-132, serine-139, cysteine-237, phenylalanine-108, glutamic acid-111 and lucine-110 with Vitamin D3. **C** 2D structure of *BDNF* (variant V66M) with vitamin D3. **D** cartoon representation of polar interaction of complex *BDNF* variant V66M with natural compound (Vit D3) by PyMOL. Yellow dotted line show polar interaction

**Fig. 3:**
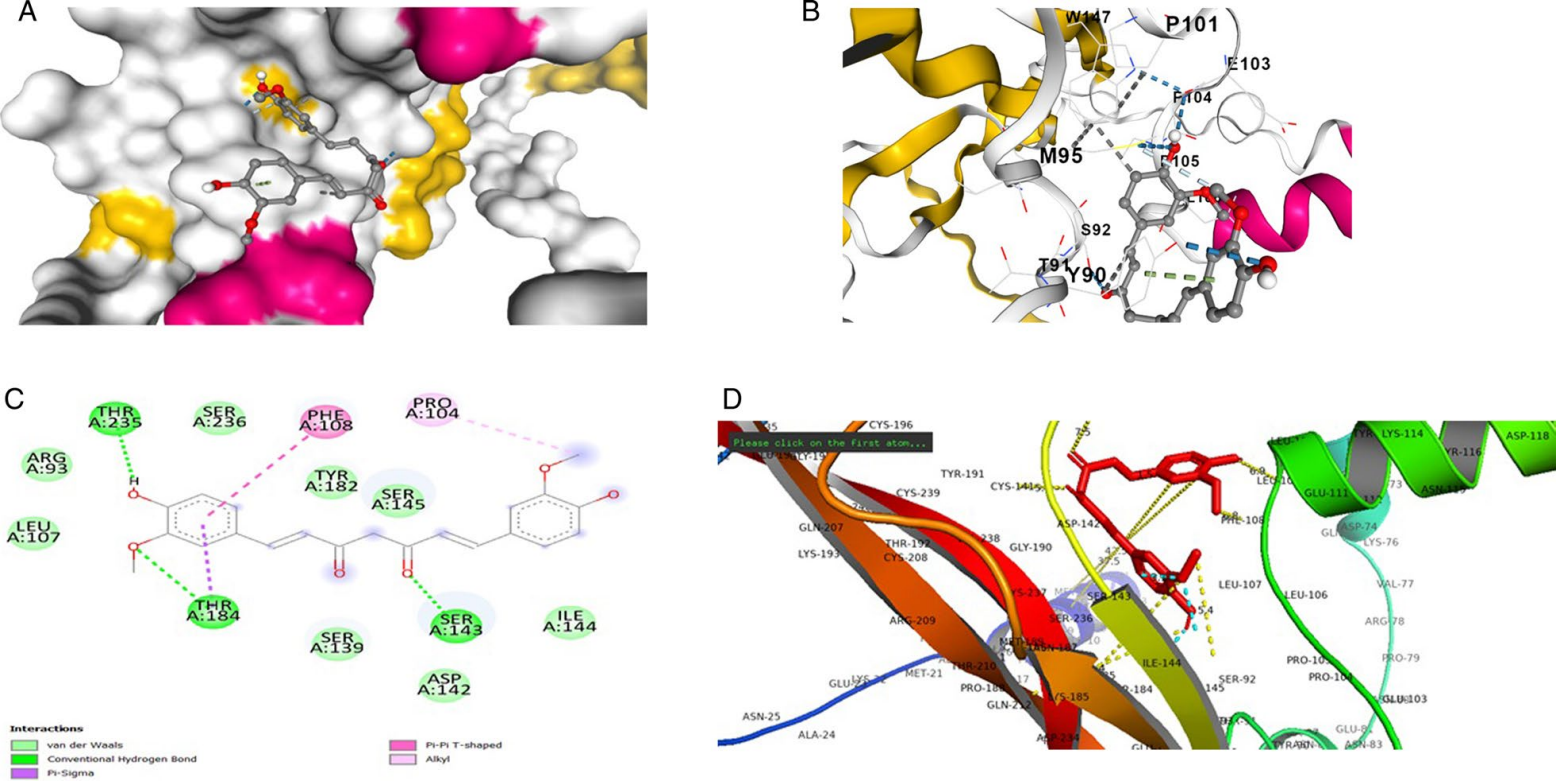
In silico modeled structure cartoon representation of complex *BDNF* variant V66M/Curcumin). **A** Spherical cartoon structure represent the binding pocket cavity interacting of residue between *BDNF* residue with Curcumin. **B** Shown interacting residue of *BDNF* muted Val-94, proline-104, cysteine, proline-101, serine-92, phenylalanine-108, glutamic acid-103, Methonine-95, Therionine-91, Tyrosin-19, Tryptophan-147 with curcumin. **C** 2D structure of *BDNF* (variant V66M) with Curcumin. **D** cartoon representation of polar interaction of complex *BDNF* variant V66M with natural compound (Curcumin) by PyMOL. Yellow dotted line show polar interaction

**Fig. 4:**
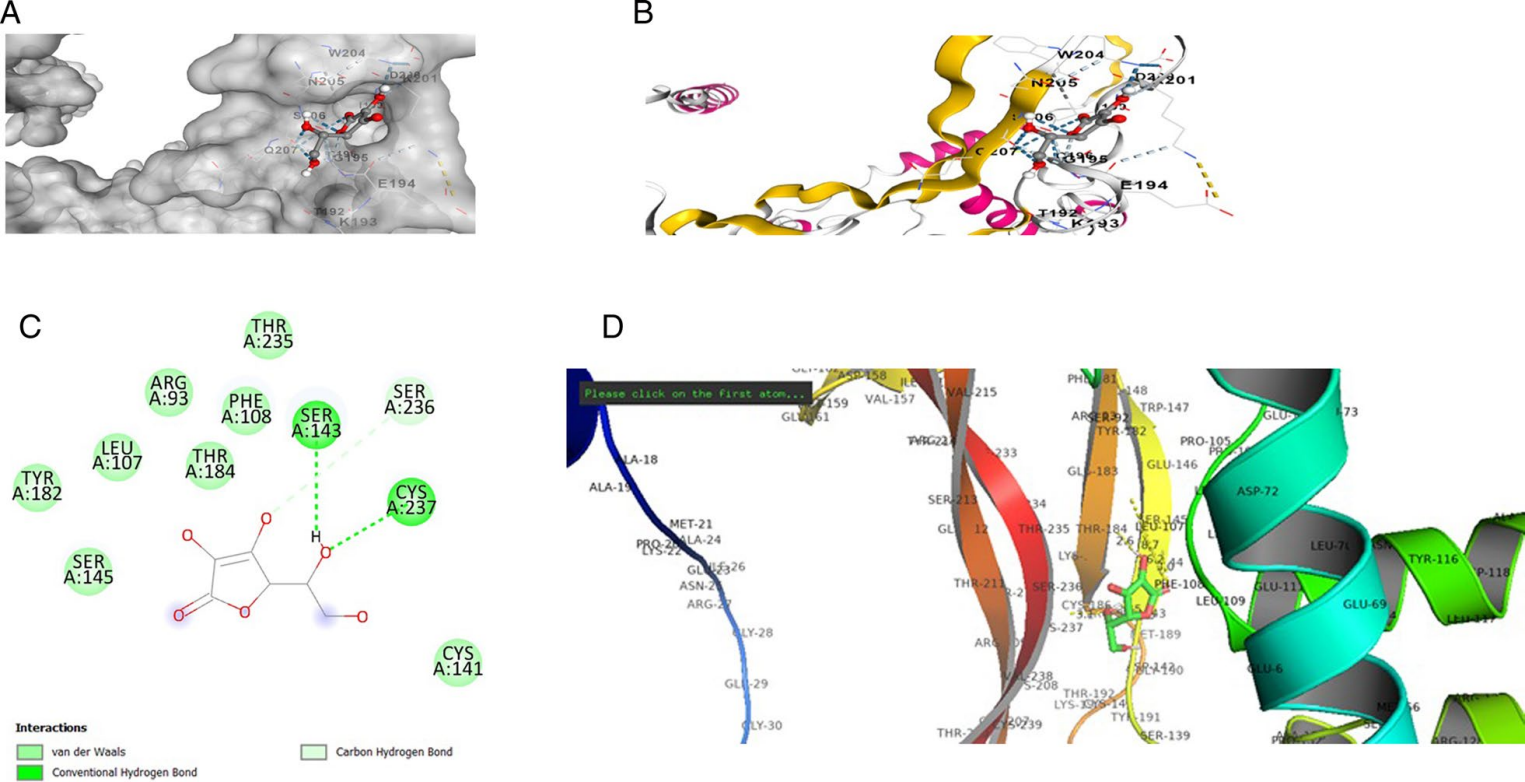
In silico modeled structure cartoon representation of complex *BDNF* variant V66M/Vitamin C. **A** Spherical cartoon structure represent the binding pocket cavity interacting of residue between *BDNF* residue with Vitamin C. **B** Shown interacting residue of *BDNF* muted Val-140, cysteine-37, proline-132, serine-139, cysteine-237, phenylalanine-108, glutamic acid-111 and lucine-110 with Vitamin C. **C** 2D structure of *BDNF* (variant V66M) with Vitamin C. **D** cartoon representation of polar interaction of complex *BDNF* variant V66M with natural compound (Vit C) by PyMOL. Yellow dotted line show polar interaction

**Fig. 5:**
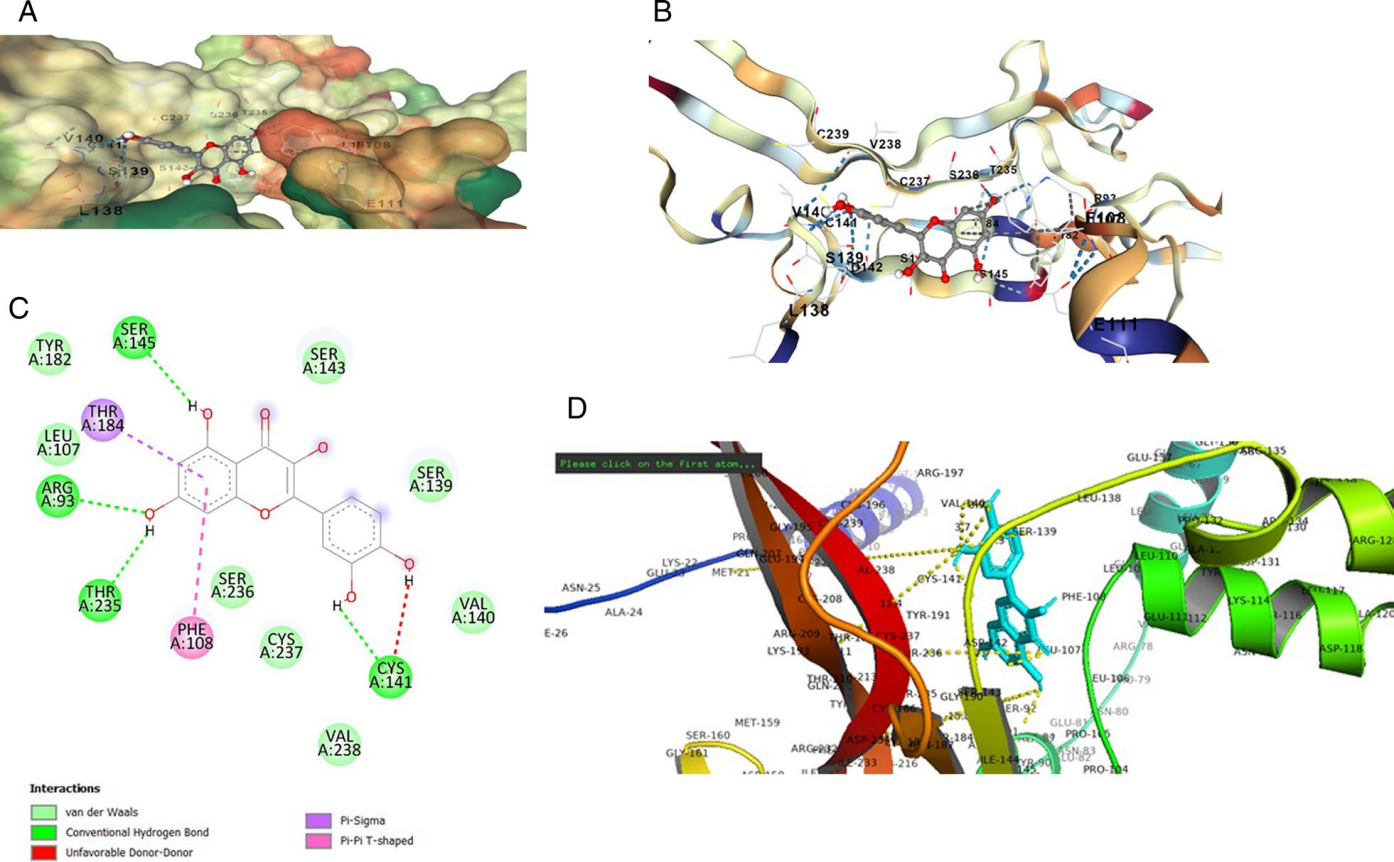
In silico modeled structure cartoon representation of complex *BDNF* variant V66M/Quercetin. **A** Spherical cartoon structure represents the binding pocket cavity interacting of residue between *BDNF* residue with quercetin. **B** Shown interacting residue of BDNF muted Val-140, proline-104, cysteine-237, valine-238, serine-139, Serine-236, lysine-107, Therionine-235, Arginine-93 with Quercetin. **C** 2D structure of *BDNF* (variant V66M) with quercetin. **D** Cartoon representation of polar interaction of complex *BDNF* variant V66M with natural compound (Quercetin) by PyMOL. Yellow dotted line show polar interaction

The *BDNF* variant V66M structure was selected as the structural interaction receptor, and the natural chemical structure was chosen as the ligand. The energy binding score, residues, and atoms produced by the contact between the receptor and ligand are characteristics used to assess structural interactions. Incorporating molecular docking techniques allowed us to screen for the selection of natural compounds. Searches of the identified drugs versus the human *BDNF* variant V66M revealed considerable suppression based on in *silico* analyses, and the observed analyses revealed trustworthy results. The four compounds that scored the highest overall from a combination of the available compounds were revealed to be novel. AUTODOCK VINA tool evaluated the binding posture of each docked ligand. The binding energy, binding affinity, and docking pose confirmation inside binding pocket were used to score docking poses. The top docking results for docked ligands are displayed in Table 1. The four most highly ranked binding energy scores for these medications against the human mutant *BDNF* (V66M) structure according to AUTODOCK VINA were − 4.5, − 6.1, − 6.7, and − 5.5 kcal/mol of Vitamin C, Curcumin, Quercetin, and Vitamin D3. We are also looking at our chosen drugs' pharmacological compatibility and molecular mechanisms (Vitamin C, Curcumin, Quercetin, and Vitamin D3) against the mutant *BDNF* structure (V66M). Analysis of the interactions between Vitamin D3 and the residues of the *BDNF* muted structure (V66M) was done using the protein–ligand contact and interaction (Fig. 2d). In the binding pocket are the lys-114, Glu-111, and Phe-101 from chain A residues of *BDNF* (V66M), which are implicated in a pi-alkyl interaction with Vitamin D3 (Fig. 2a–c). The binding pocket surrounding the *BDNF* muted structure (V66M) is revealed by the interaction 2D graph (Fig. 2d), and it contains amino acids like Val-140, cysteine-37, proline-132, serine-139, cysteine-237, phenylalanine-108, glutamic acid-111, and Lucien from chain A. The residues of the BDNF mutated structure (V66M) were examined using the analysis of the protein–ligand contact 2D graphs and interactions (Fig. 3d). Chain A: Thr-235, Thr-184, and Ser-143 are significant *BDNF* (V66M) residues that are situated inside the binding pocket and participate in a three hydrogen bonding and one pi–pi interaction with curcumin. The binding pocket surrounding essential *BDNF*-muted structure (V66M) amino acids such as Val-94, proline-104, cysteine, proline-101, serine-92, phenylalanine-108, glutamic acid-103, Methonine-95, Therionine-91, Tyrosin-19, and Tryptophan-147 from chain A were involved, according to the interaction 2D graph (Fig. 3d). The curcumin can impede *BDNF* activity by firmly fitting within the binding pocket of the molecule (V66M). Analysis of the protein–ligand contacts in 2D graphs and the interaction between vitamin C and the residues of the *BDNF* mutated structure (V66M) were performed (Fig. 4d). Ser-143 and Cys-237 from chain A are crucial *BDNF* (V66M) residues situated inside the binding pocket and are implicated in two hydrogen bonds with vitamin C (Fig. 4a–c). The interaction 2D graph (Fig. 3d) shows that the binding pocket surrounding the essential *BDNF* muted structure (V66M) is involved in the interaction loop with vitamin C and Other amino acids from chain A, including cysteine-37, proline-132, serine-139, cysteine-237, phenylalanine-108, glutamic acid-111, and lucine-110, were also involved. Quercetin and the residues of the *BDNF* mutated structure (V66M) were examined using the analysis of the protein–ligand contact 2D graphs and interactions (Fig. 5d). Ser-145, Arg-93, Thr-235, and Cys-141 are significant *BDNF* (V66M) residues in chain A that are located inside the binding pocket and participate in four-hydrogen bond and two pi-pi bond interactions with quercetin (Fig. 5a–c). The binding pocket surrounding essential *BDNF*-muted structure (V66M) amino acids such as proline-104, cysteine-237, valine-238, serine-139, serine-236, lysine-107, therionine-235, and arginine-93 from chain A are involved according to the interaction 2D graph. Quercetin exhibited a significant inhibitory interaction potential to block *BDNF* (V66M), fitting well within the protein's binding pocket (Table 1 and Figs. 2, 3, 4 and 5a–d).

**Table 1:** Molecular docking alignment of different natural compounds with *BDNF* variant V66M complexes was analyzed

Protein and Ligand properties	*BDNF* variant (V66M)/Vitamin D3	*BDNF* variant (V66M)/Curcumin	*BDNF* variant (V66M)/Vitamin C	*BDNF* variant (V66M)/Quercetin
Total Binding energy	− 5.5 kj/mol	− 6.1 kj/mol	− 4.5 kj/mol	− 6.7 kj/mol
H-bond	H–R	H–O	H–O	H–O
Residue	LYS-114, PRO-132, PHE-108	THR-235, THR-184, Serine-143	SER-143, CYS-237	SER-145, THR-235, CYS-141
H-bond energy kcal/mol	− 3.2, − 2.6, − 2.1 kcal/mol	− 1.8, − 1.5, − 2.5 kcal/mol	− 1.7, − 2.8 kcal/mol	− 1.9, − 1.8, − 2.2 kcal/mol
Interacting residue of BDNF variant (V66M)	Val-140, cysteine-37, proline-132, serine-139, cysteine-237, phenylalanine-108, glutamic acid-111 and lucine-110	Val-94, proline-104, cysteine, proline-101, serine-92, phenylalanine-108, glutamic acid-103, Methonine-95, Therionine-91, Tyrosin-19, Tryptophan-147	Serine-92, glutamic acid-194, Glutamine-207, aspartic acid-200, Glycine-195, lysine-193, asparagine-205, Methonine-95, Tyrosin-19,	Val-140, proline-104, cysteine-237, valine-238, serine-139, Serine-236, lysine-107, Therionine-235, Arginine-93,

### Pre-clinical testing of drug analyses/toxicity analyses

**Fig. 6:**
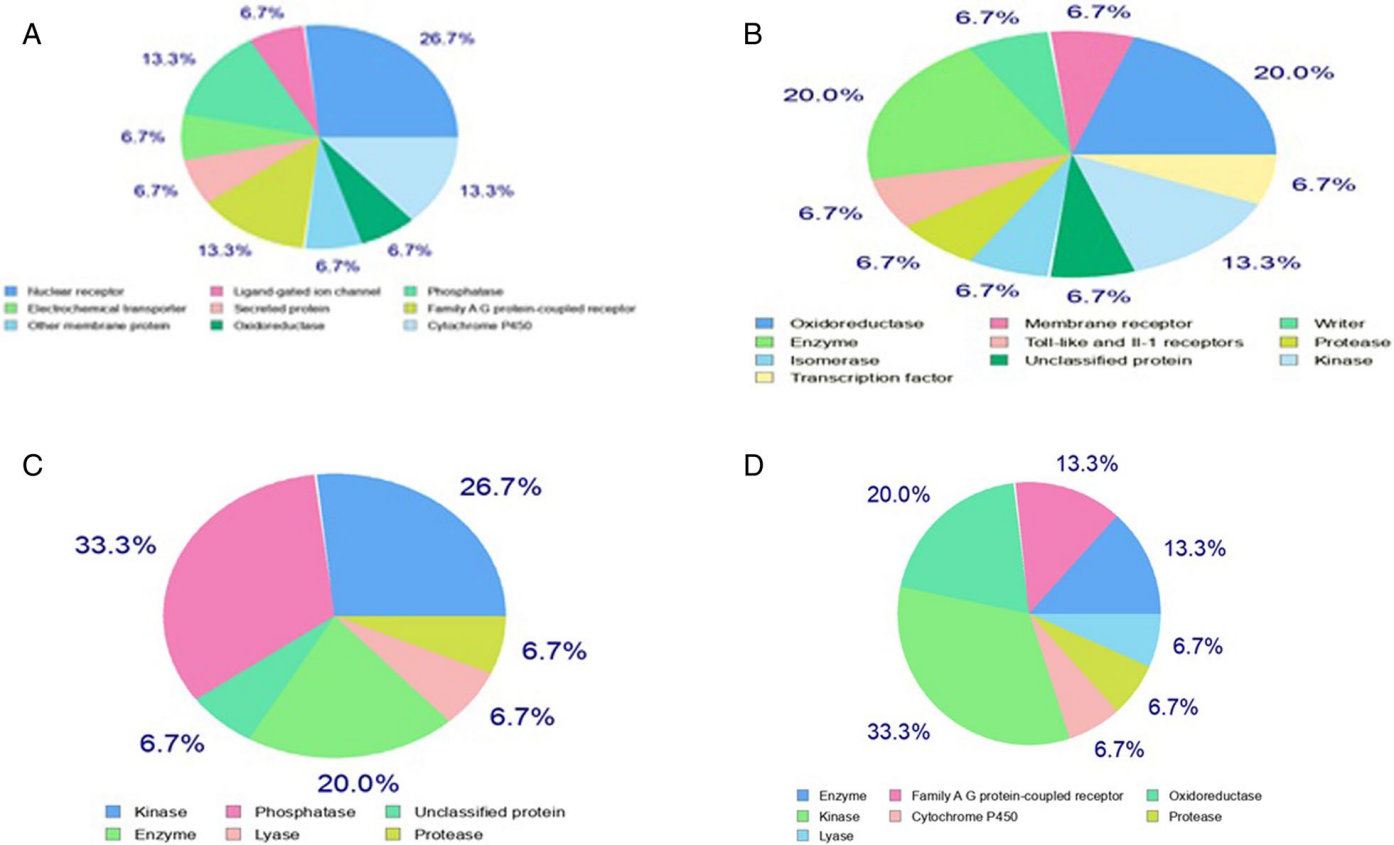
In silico modeled show pi-diagram of natural compound ligand (Vit D3, Curcumin, Vit C, and Quercetin) by Swiss target prediction tool. Indicate target prediction. Ligand highly hits different targets. **A** Vitamin D3 target 26.7% nuclear receptor. The muted *BDNF* gene, interactions of vitamin D3 with GPCRs, and associated signaling pathways may be of interest. **B** Curcumin target 20% Oxidoreductase and enzyme. curcumin interacts with oxidoreductases and kinases in the context of compensatory mechanisms, or by modifying the BDNF-related effects caused by mutations **C** Vitamin C highly target 33.3% phosphatase. Vitamin C interacts with phosphatases and kinases in the context of compensatory mechanisms or moderating effects of BDNF-related mutations **D** Quercetin target 33.3% oxidoreductase. BDNF activity is impaired, the capacity of quercetin to interact with these enzymes may affect neuroprotection

Algorithm Swiss ADMET was used to examine the top-ranked four compounds' ADMET characteristics and pharmacological properties. According to Table 2, the chosen compounds display significant biological features, see Fig. 6a–d.

**Table 2:** To analyze preclinical drug testing of four different natural compounds

Ligands properties	*BDNF* variant (V66M)/Vitamin D3	*BDNF* variant (V66M)/Curcumin	*BDNF* variant (V66M)/Vitamin C	*BDNF* variant (V66M)/Quercetin
*Physiochemical properties*	*–*	*–*	*–*	*–*
Molecular weight g/mol	384.64 g/mol	368.38 g/mol	176.12 g/mol	302.24 g/mol
Number of heavy atoms	28	27	12	22
Hydrogen bond acceptor	11	6	6	3
Hydrogen bond donor	1	2	4	5
Rotatable bonds	6	8	2	1
Fractional Csp3	0.78	0.14	0.50	0
Molar refractivity	125.04	102.80	35.12	78.03
TPSA	20.23 Å2	93.06 Å^2^	107.22	131.36 Å^2^
*Drug solubility*				
Log S	0.20	− 3.94	0.23	− 3.16
*Druglikness*				
Lipinski	No	No	No	Yes
Bioviablity score	0.08	0.55	0.55	0.59
*Pharmacodynamics*				
Human intestinal absorption (probability)	No	High	High	High
Blood brain barrier (probability)	No	No	No	No
CYP450 2D6	No	No	No	No
Log Kp (skin (permeate)	− 6.3 cm/s^2^	− 6.28 cm/s	− 8.54 cm/s	− 7.05 cm/s
*Medicinal chemistry*				
PAINS	0 alert	0 alert	0 alert	1 alert
Brenk	0 alert	2 alerts	0 alert	1 alert
Leadlikness	No	Yes	No	Yes

### Molecular simulation of docking compounds study

**Fig. 7:**
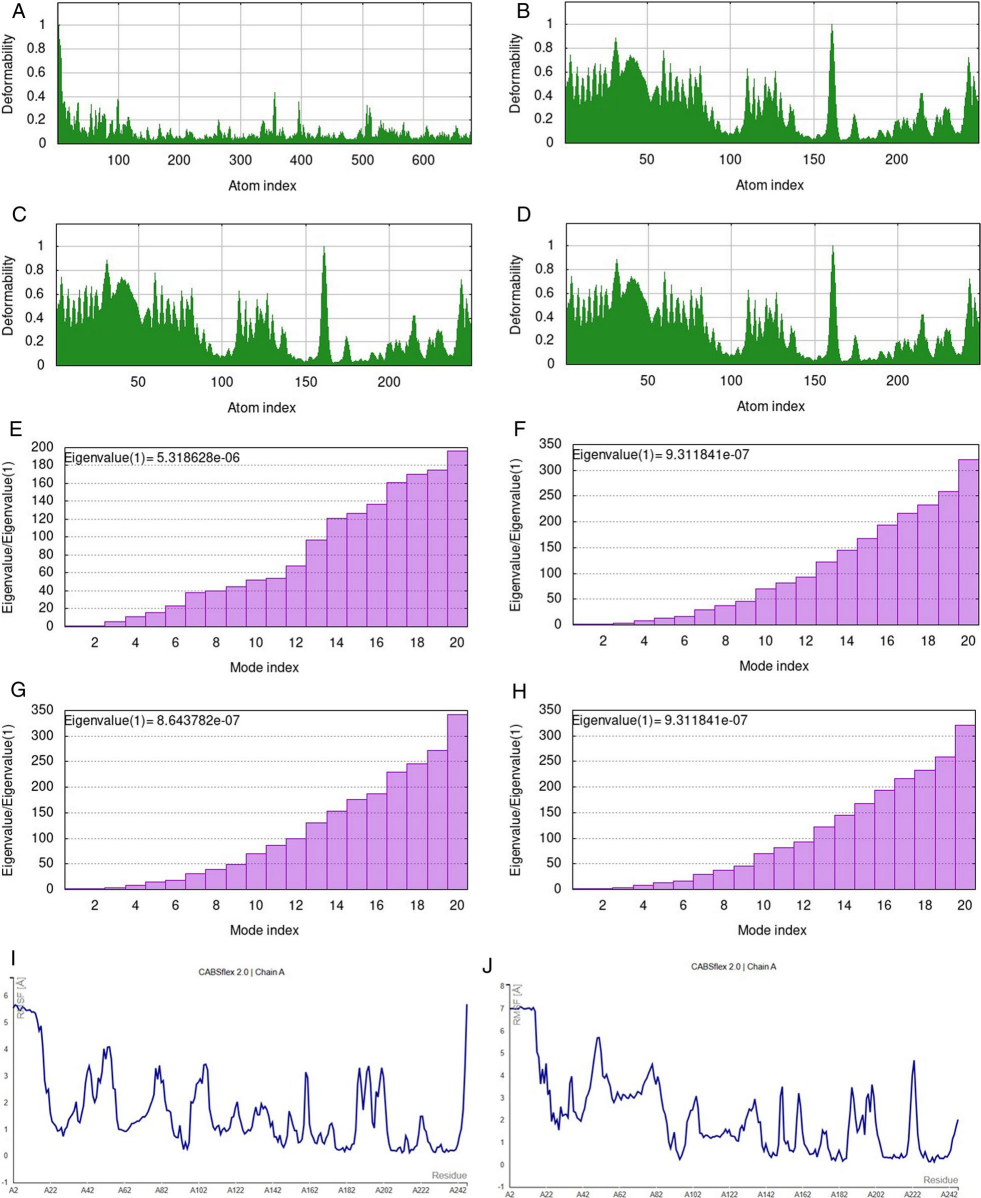

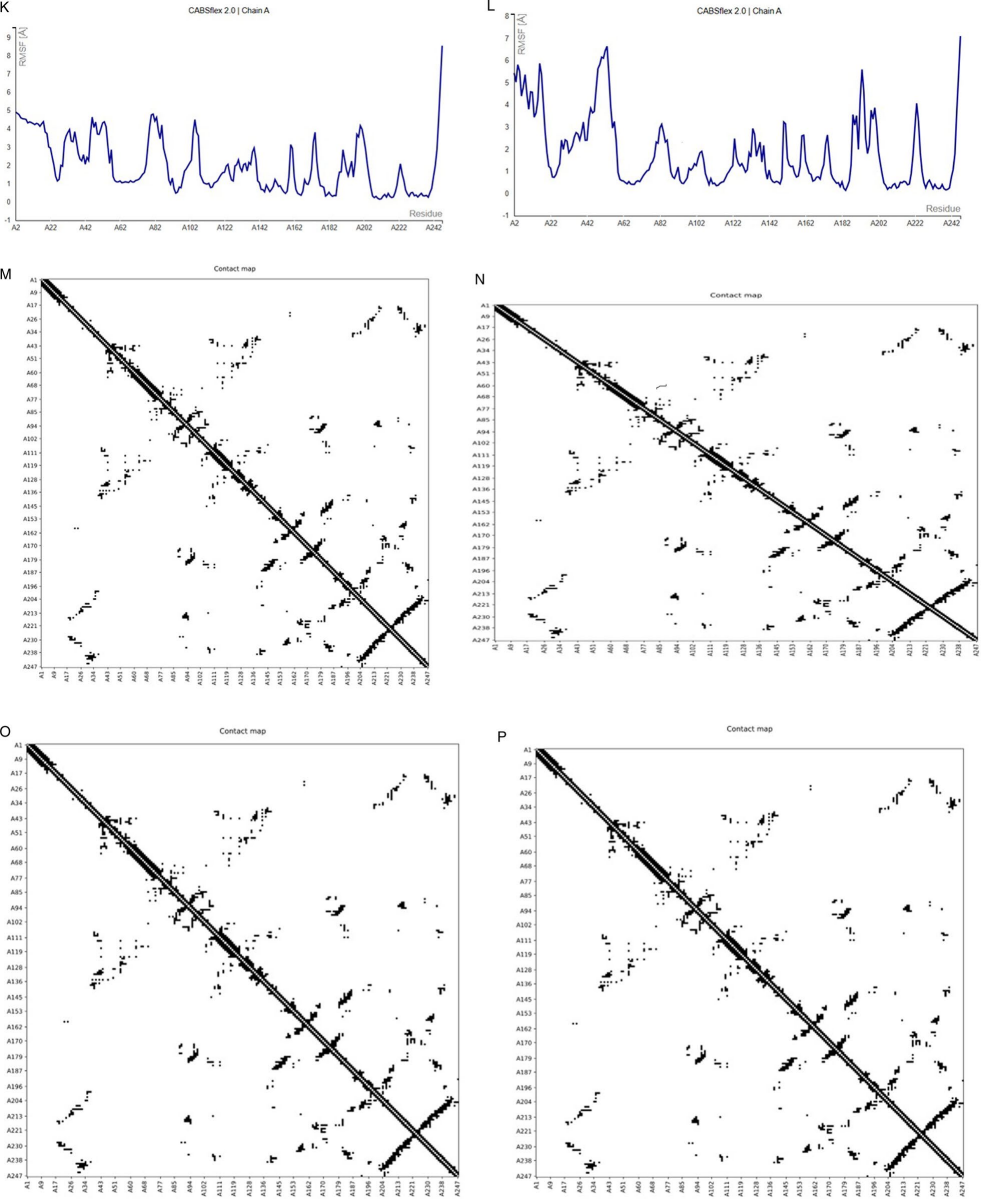
MD simulation modeled structural complex. **A**–**D** The deformability is to indicate that residue interact with each other through deformation between protein with different natural compounds therapy against BDNF muted structure. **E**–**H** represent Eigenvalue (1) of complexes. Eigenvalues in NMA are not directly linked to the stiffness or hardness of a molecule; instead, they are associated with the energy associated with vibrational modes and deformation. **I**–**L** represent RMSF graph of peptide-ligand complex (Vitamin D3, Curcumin, Vitamin C, Quercetin). **M**–**P** represent dot map indicate strong residue-residue interaction amonf residue of BDNF (V66M) target peptide

To perform Normal Mode Analysis (NMA) in internal coordinates (IC) on the atomic structures of peptides and natural compounds, iMODs is a versatile toolkit. iMODs server determines the type of contact and the distance in (C) between the interacting residues. As a result, vibrational analyses, motion animations, and simulations at different resolution levels are made simple, see Fig. 7(A–H). Figure 7(I–L) presents the fluctuation plot of the simulation (RMSF), whereas Fig. 7(M–P) shows the residue contact map.

## Discussion

The single nucleotide polymorphism Val66Met affects the processing and secretion of *BDNF*, potentially leading to decreased availability and impaired synaptic plasticity (Yao et al. 2020). There may be a link between the Val66Met variation and neurological conditions like schizophrenia and Alzheimer's disease, according to certain studies (Mohammadi et al. 2018).

Our in silico study found that AUTODOC VINA, BIOVIA Discovery Studio, PyMOL, and CB-dock server performed complete molecular docking of *BDNF* (Variant V66M) with Vitamin D, Curcumin, Vitamin C and Quercetin compounds. CB-Dock is the first cavity detection-guided blind docking tool designed with AutoDock Vina. Among many popular Vina-based tools CB-Dock outperforms other state-of-the-art blind docking tools in predicting binding sites and binding conformations. This performance is attributed to curvature-based cavity detection, which precisely narrows the docking space and the optimized parameters for AutoDock Vina. After molecular docking were examinations of the least binding energies for *BDNF* (Variant V66M) with Vitamin D3 (Paolacci et al. 2020), Curcumin (Jin et al. 2022), Vitamin C (Tveden-Nyborg 2021) and Quercetin (Zhang et al. 2020). The chosen ligands that interacted well with the receptor protein are shown in Fig. 1a–d. The alignment produces the energy binding-score to evaluate the molecules' structural interaction (Opo et al. 2021; Wu et al. 2018).

For cavities, CB-Dock looks for concave surfaces. The interacting residues of protein *BDNF* variant V66M in chain A: Residues: Val-94, proline-104, cysteine, proline-101, serine-92, phenylalanine-108, glutamic acid-103, Methonine-95, Therionine-91, Tyrosin-19, Tryptophan-147 with Curcumin were shown − 6.5 kcal/mol binding energy. The interacting residue of *BDNF* (Variant V66M) receptor in Chain A. Residue: Serine-92, glutamic acid-194, Glutamine-207, aspartic acid-200, Glycine-195, lysine-193, asparagine-205, Methonine-95, Tyrosin-19 with Vitamin C show − 4.5 kcal/mol binding energy. The interacting residue of *BDNF* muted gene in chain A. Residue: Val-140, proline-104, cysteine-237, valine-238, serine-139, Serine-236, lysine-107, Therionine-235, Arginine-93, with Querecitin compound and show − 6.7 kcal/mol binding energy, see Table 1. The docking results revealed that quercetin is a more potent ligand than the other three ligands that bind more strongly with BDNF (V66M) in the mutated structure, halting the process of BDNF (V66M) that leads to stress. This is because four strong hydrogen bonds are formed between the residues of BDNF (V66M) and quercetin, in addition to a pi–pi interaction. In comparison, the interaction of the other three ligands with the BDNF mutated structure (V66M) resulted in a smaller number of hydrogen bonds. These algorithms developed thorough models of the Protein ligands complex showing the binding energy results (Emamian et al. 2019).

Pre-clinical drug testing or Toxicology analyses were conducted to learn more about the chosen ligands by Swiss ADMET server (Sahu et al. 2022). The Based on physiochemical properties, the chemical structures of the chosen compounds (Vitamin D3, Curcumin, Vitamin C, and Quercetin) demonstrated beneficial drug-like qualities, and their molecular weight 384.64 g/mol, 368.38 g/mol, 176.12 g/mol, and 302.24 g/mol. A drug-likeness assessment measures the oral bioavailability of a therapeutic molecule. It is described as the amount and pace at which a medicine taken orally enters the bloodstream and reaches the intended target areas. The ligands were screened for drug-likeness using Lipinski's Rule of Five. The compound has completed the evaluation and is regarded as orally bioavailable. According to Lipinski's rule of five, ligands with MW > 500 and HBA > 10 may not have excellent oral absorption because they break two parameters. Our finding shows that the quercetin ligands have no violation and three hydrogen bond acceptor (HBA = 3), which indicate good oral absorption in Table 2.

In silico analysis, according to Lipinski's criterion, the chemical structures of the selected compounds showed useful drug-like properties that increased oral bioavailability (Chandran et al. 2022; Pandi et al. 2022). Oral bioavailability is related to the active form of a medicinal compound that makes it unmodified for systemic circulation. Drugs were deemed orally bioavailable if their oral bioavailability scores were > 0.1. All ligands' bioavailability values ranged from 0.08 to 0.55 to 0.56 and 0.59, showing oral bioavailability. However, following Lipinski's rule of five, the ligand quercetin showed the best oral bioavailability, with a score of 0.59. The term "blood–brain barrier" (BBB) also refers to the brain's microvascular endothelial cell layer, which isolates the brain from blood flow and blocks the entry of toxins into the central nervous system (CNS). The BBB permeability results in Table 2 indicate that none of the ligands could cross the BBB. This might be useful because they may not have any negative effects on the CNS when used as medications.

Pharmacokinetics examines the behavior of a medicinal chemical in the body, specifically about its absorption (A), distribution (D), metabolism (M), and excretion (E). In silico, GIT, blood barrier penetration (Tian et al. 2022), skin permeability, aqueous solubility [logS] (Dulsat et al. 2023), acute oral toxicity, and cytochrome P450 2D6 inhibition (Tahir et al. 2019), were used to calculate the ADMET properties of the chosen compounds (Rahman et al. 2021). Therefore, before the clinical stage, drug candidate profiling is a practical drug discovery technique. All the ligands were found to have the potential to be ingested.

One of the most critical phases in contemporary drug discovery is the assessment of the metabolism and biotransformation of therapeutic candidates. The cytochrome P450 (CYP450) monooxygenase family plays a significant role in this process. Because of bioaccumulation, the inhibition of CYP450 enzymes by a medicinal substance may result in toxicological profiles and poor bioavailability. According to the findings in Table 2, it is possible that they metabolized, biotransformed, and eliminated from the body. It's important to remember that Curcumin, and Vitamin D3 (Kardan et al. 2023), Vitamin C, and Quercetin bioavailability can change based on the precise formulation and dosage (Riva et al. 2019).

Various in silico toxicity tests were performed to assess the adaptability of the selected drugs. The right dosing schedules and predictions of drug behavior in the body depend on an understanding of pharmacokinetics (Migliorati et al. 2022). According to the pharmacodynamics results, our compounds did not respond to different cytochromes and blood–brain barriers and regulated high intestinal absorption. These drugs couldn't permeate the skin (Chandrasekaran et al. 2018). Our finding indicates that Quercetin ligands have an excellent therapeutic effect on BDNF variant (V66M) without cause of toxicity according to Lipinski's rule.

In silico modeled show pi-diagram of natural compound ligands (Vit D3, Curcumin, Vit C, and Quercetin) by Swiss target prediction tool (Patil and Rohane 2021). Ligands hit different targets. Vitamin D3 targets 26.7% nuclear receptor, 13.3% cytochrome p450, and other G couple protein. The interaction between vitamin D3 and nuclear receptors may affect the expression of genes linked to *BDNF* signalling, brain health, and other physiological functions when *BDN*F is a muted structure. Due to their putative functions in regulating neuronal function, the muted *BDNF* gene, interactions of vitamin D3 with GPCRs, and associated signalling pathways (Cui et al. 2017). Curcumin targets 20% oxidoreductase and 13.3% kinase. Curcumin has been studied for its potential to inhibit acetylcholinesterase, an enzyme linked to neurodegenerative diseases, including Alzheimer's disease, in the context of neuroprotection. This restriction may preserve the levels of acetylcholine in the brain. However, interactions of curcumin with enzymes and oxidoreductases may have therapeutic consequences. Alterations in *BDNF* expression or function may result from *BDNF* gene mutations, which may affect brain and neuronal health. It may be important to consider how curcumin interacts with oxidoreductases and kinases in the context of compensatory mechanisms, or by modifying the *BDNF*-related effects caused by mutations (Jabir et al. 2018). Vitamin C targets 33.3% phosphatase and kinase 27%. *BDNF* signalling pathways, crucial for brain development, synaptic plasticity, and cell survival, require kinase activity. How vitamin C interacts with kinases may affect the regulation of *BDNF*-related functions. Alterations in *BDNF* expression or function may result from *BDNF* gene mutations, which may affect brain and neuronal health. It may be important to consider vitamin C interacts with phosphatases and kinases in the context of compensatory mechanisms or moderating effects of *BDNF*-related mutations (van Woerkom and Treatment 2017). Quercetin targets 33.3% of oxidoreductase, 20% of kinase, and oxidoreductase. Quercetin's interactions with kinases may impact the *BDNF* gene and BDNF signalling pathways. The neuronal growth, survival, and plasticity processes depend on *BDNF* signalling, which is influenced by kinase activity. The expression or function of *BDNF* may be altered if the *BDNF* gene harbors a mutation. Numerous neurological and behavioral diseases have been linked to BDNF mutations. The modulation of compensatory mechanisms that affect *BDNF* signaling or neuronal health by the interactions of quercetin with oxidoreductases and kinases may be pertinent in the context of a silenced *BDNF* gene. In situations where *BDNF* activity is impaired, the capacity of quercetin to interact with these enzymes may affect neuroprotection and brain health (Grewal et al. 2021) see in Fig. 6a–d.

The *BDNF* muted structure (Variant V66M) underwent thorough computational investigations in the current work, followed by evaluations using molecular dynamic simulations NMA (Chmiela et al. 2018). The compounds under evaluation may be utilized for tackling the *BDNF*-muted structure (Variant V66M), as determined by comparing studies using molecular docking and molecular dynamic simulations NMA. In *silico* analysis, NMA mobility of protein with ligand complex highlight side chain residue interacts with the other residue, leading stiffness of four protein/compound complexes using iMOD serevr. The deformability analysis of Curcumin and Quercetin ligands indicates minimal distortion in the complex, as indicated by the hinges, compared to other ligands, including Vitamin C and Vitamin D3. The chain of high hinge location regions showed more excellent deformability between proteins with different natural compound therapies against *BDNF* muted structures. The hinges of our protein with varying complexes of compound were similar to the experimental PDB results (Fig. 7a–d). Additionally, a higher eigenvalue value 9.311841e-07 was observed, which shows the energy needed to deform the complex of curcumin and quercetin with BDNF (V66M), indicating its stability. The more complex properties of molecular stiffness are influenced by several factors, including the bond strength and structural characteristics shown in Fig. 7(e–h) (Ireoluwa Yinka et al. 2020).

The results of the MD study presented as a plot of RMSF against the protein residues of BDNF (V66M) revealed that most of the active residues of the target macromolecule fluctuated stably around less than 3 A˚, an indication that the protein structure is stable and does not diverge significantly from its initial structure. The protein–ligand complex was subjected to MD simulation analysis to ascertain the stability of the interaction between the ligands Vitamin D3, Curcumin, Vitamin C, and Quercetin with the target protein BDNF (V66M) by fluctuation plot (RMSF). Structural alterations in the protein may be the cause of the considerable divergence observed for Vitamin D3 residues A5 (5.2 Å), A3 (5.6 Å), A7 (5.7 Å), and A247 (5.6 Å). The structural alterations in the protein may be the cause of the considerable divergence observed for Curcumin residues A2 (6.9 Å), A8 (7.0 Å), A15 (7.3 Å), A51 (5.6 Å), and A223 (4.6 Å). Structural alterations in the protein may be the cause of the considerable divergence observed for the Vitamin C residues A2 (4.9 Å), A7 (4.6 Å), A9 (4.8 Å), A56 (4.6 Å), and A80 (4.8 Å). However, it was discovered that the other protein–ligand complexes diverged significantly from their original structures. But Quercetin with BDNF (V66M) residues A18 (5.8 Å), A52 (6.4 Å), and A247 (7.1 Å) showed considerable divergence, which may be the result of structural changes in the protein. When the results of the MD study were plotted against the protein residues of BDNF (V66M) with quercetin, they showed that the majority of the active residues of the target macromolecule fluctuated steadily around less than 3 Å, demonstrating that the protein structure is stable and does not significantly deviate from its starting structure. The various visuals of the model serve as evidence of the macromolecule's structural variety (Fig. 7i–l). The "contact map" in figure also provides a thorough perspective of the protein's residue-residue interaction pattern. Each dot on the map represents an interaction between a pair of residues, and the map's color determines how frequently these interactions occur. On a scale of 1.0, the prevalence of deep dark colors shows that the BDNF (V66M) target residues have many strong residue interactions see Fig. 7m–p. Finally, our simulation results of structural models can be validated using NMA and PDB data consistency. As determined by MD simulation, the most stable compound has a dynamic binding of the ligand (quercetin) to the target protein BDNF (V66M).

In this particular work, comprehensive computational approaches involving high-throughput capabilities screening, molecular docking research, and molecular dynamic simulations were applied (Islam et al. 2023). The results of the molecular docking studies suggested that Quercetin compounds might be effective against the *BDNF* (Variant V66M). After thorough in silico investigations from the chosen libraries, it was interestingly found that the reported compounds together displayed the lowest binding energy. The compounds found in the present investigation demonstrated a propensity for good therapy against *BDNF* muted structure (variant V66M).

In conclusion, the current study explores the inhibitory effect of four natural compounds, Vitamin D, Curcumin, Vitamin C, and Quercetin, on the human muted structure of *BDNF* variant (V66M). The final selection of the potential *BDNF* variant (V66M) inhibitor was based on docking, pre-clinical toxic analysis, and Molecular Dynamics Simulations. As determined by MD simulation, the most stable compound has a dynamic binding of the ligand (Quercetin) to the target protein BDNF (V66M). The molecular docking and simulation results showed Quercetin has a more potent inhibitory binding effect on *BDNF* variant (V66M) and can be more suitable as anti-stress agent. The chosen ligand Quercetin was subjected to in *silico* ADMET analysis, which showed favorable pharmacokinetic and toxicological profiles.

### Limitations and future directions

Due to limited financial resources, we regret to report our inability to conduct the wet lab experiment. As a future recommendation, we propose investigating this variant within the Pakistani population to corroborate the in silico findings. Additionally, to explore the potential involvement of the BDNF variant (V66M) in psychological disorders.

## Data Availability

The produced, collected or generated during study has been given in the manuscript file.
